# Clustering of Parkinson subtypes reveals strong influence of DRD2 polymorphism and gender

**DOI:** 10.1038/s41598-022-09657-0

**Published:** 2022-04-11

**Authors:** Esther Annegret Pelzer, Sophie Stürmer, Delia-Lisa Feis, Corina Melzer, Frank Schwartz, Marcel Scharge, Carsten Eggers, Marc Tittgemeyer, Lars Timmermann

**Affiliations:** 1 Max Planck Institute for Metabolism Research, Translational Neurocircuitry Group, Gleulerstrasse 50, 50931 Cologne, Germany; 2 Department of Neurology, University Hospital Cologne, Cologne, Germany; 3 Department of Neurology, University Hospital Marburg, Marburg, Germany; 4 Excellence Cluster on Cellular Stress Responses in Aging Associated Diseases (CECAD), University of Cologne, Cologne, Germany; 5 Department of Psychiatry, University Hospital Cologne, Cologne, Germany

**Keywords:** Parkinson's disease, Machine learning

## Abstract

Most classification approaches for idiopathic Parkinson’s disease subtypes primarily focus on motor and non-motor symptoms. Besides these characteristics, other features, including gender or genetic polymorphism of dopamine receptors are potential factors influencing the disease’s phenotype. By utilizing a kmeans-clustering algorithm we were able to identify three subgroups mainly characterized by gender, DRD2 Taq1A (rs1800497) polymorphism—associated with changes in dopamine signaling in the brain—and disease progression. A subsequent regression analysis of these subgroups further suggests an influence of their characteristics on the daily levodopa dosage, an indicator for medication response. These findings could promote further enhancements in individualized therapies for idiopathic Parkinson’s disease.

## Introduction

Parkinson’s disease (PD) is next to Alzheimer’s disease one of the most common neurodegenerative disorders^1^. It manifests with heterogeneous courses of clinical symptoms^2^ in the motor and non-motor domain. To better understand the underlying pathophysiology of the disease and its characteristic, PD patients are mostly differentiated based on their motor symptoms into akinetic-rigidic, mixed-type and tremor-dominant subtype^3^. Such characterizations aim to optimally fit therapeutic regimes to patients and therefore reduce undesired side effects.

So far, the classification of subtypes and their subsequent therapeutic decisions are mostly driven by empirical impressions of the physician. These are predominantly determined by motor scores such as the UPDRS^4^ or the Hoehn and Yahr Scale^5^. In the last decades further knowledge about Parkinson’s disease was gained, particularly with respect to the diagnostic importance of non-motor symptoms^6^.

A common technique for subdividing data into unknown groups is cluster analysis. This unsupervised learning approach aims to separate data items (e.g. patients) into groups of similar characteristics. Such cluster analyses (CA)^7^ have been extensively used to better identify PD subtypes in the motor and non-motor domain independently from the clinical expertise of the doctor (see Refs.^8–12^). These methods comprise the detection of new subtypes based on pre-defined clinical characteristics such as age at onset, motor phenotype, non-motor symptoms or disease progression. However, all studies highly vary in the final number of characterized clusters, sample size, inclusion criteria as well as included variables, which limits comparability.

Previous studies suggest, that beside motor and non-motor phenomena, other features could play a crucial role in the development of parkinsonian symptoms. In particular, the following measures emerge: (1) Gender differences: PD occurs 1.5 times more often in men than in women^13^. Additionally, men show an earlier disease onset and have more diverse clinical profiles than women^14^. (2) Gene polymorphism: Likewise, DRD2 Taq1A (rs1800497) and DRD3 Ser9Gly (rs6208) polymorphism might be of great importance when delineating PD subtypes^15^. An association of receptor polymorphisms—as disease causing variants—has been reported for Parkinson’s disease^16,17^. An influence of these polymorphisms on medication response and occurrence of side effects in individual patients is part of ongoing research^18–22^.

In order to proof our hypothesis of an influence of dopamine receptor polymorphisms and gender on the daily needed levodopa dosage (LED), we wanted to explore their capability of revealing new subtypes of Parkinson disease. Additionally, we were also interested in the predictive power of these subtypes for the levodopa dosage. Therefore, we utilize a kmeans-clustering approach on our patient cohort and subsequently perform multiple linear regressions for each of the delineated clusters to determine their predictive power of modeling LED.

## Methods

All patients with Parkinson’s disease or the legally authorized representative of the patients gave written informed consent. Approval of the ethics commission of Cologne University’s Faculty of Medicine (Nr.: 12-268) was obtained. The study was performed in accordance with the Declaration of Helsinki. Detailed patient information, methods and any associated references are available in the supplementary information of this paper.

## Results

**Figure 1:**
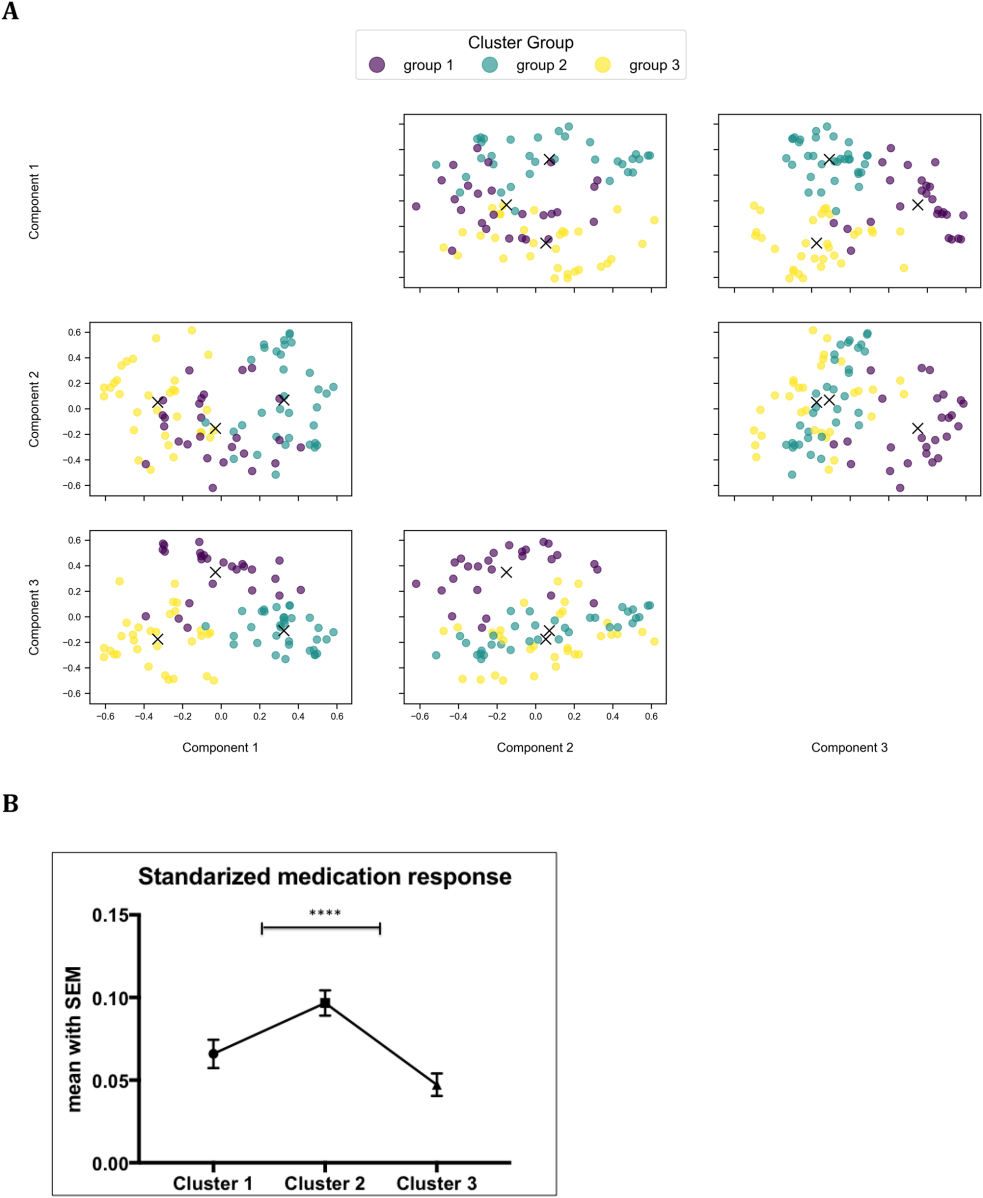
Overview of key results. (**A**) Results of kmeans-clustering with resulting three distinct groups (labeled in yellow, green and purple) exemplarily for the first three components (out of 8 components). (**B**) Cluster 2 showed the best medication response with a significant difference to cluster 1 and 3. Differences are marked at significance level of p < 0.0001 (****).

We performed kmeans-clustering for 91 patients with idiopathic PD (iPD) including the following variables: DRD2 Taq1A (rs1800497) and DRD3 Ser9Gly (rs6208) polymorphisms, standardized medication response, the age at onset, symptom onset side, a score relating the tremor/akinetic-rigidity score, disease progression in the OFF state, and gender (cf. Table 1). After validating the clustering results for 2 to 10 clusters (cf. Supplementary Fig. S2), the optimal solution results in three distinct groups of patients (cf. Fig. 1a). Post-hoc analyses of all input variables for the allocated groups revealed significant differences in patient’s gender (p < 0.0001), their individual standardized medication response (p < 0.0001), their DRD2 Taq1A (r800497) polymorphism (p < 0.0001), disease progression OFF (p < 0.0001) and symptom onset side (p = 0.017). All other variables showed no significant group differences (cf. Supplementary Table S4).

**Table 1:** Patient demographics.

Number of patients	91
Sex men/women (% men)	62 (68%)
Age at onset (years)	58 (± 10)
Symptom onset left (% left)	44 (48%)
Disease progression ‘off’	5 (± 2)
Medication response	0.41 (± 0.2)
Tremor-/akinetic-rigid-score (T-/AR-score)	0.3 (± 0.5)
DRD2 Taq1A (rs1800497) Risk type (A/A or A/G)	27 (30%)
DRD3 Ser9Gly (rs6208) Risk type (C/C or C/T)	36 (40%)

Variables included in cluster analysis. Mean (SD) in continuous variables; absolute numbers and percentage in dichotomous variables.

In summary, the three clusters could be characterized mainly by gender and DRD2 Taq1A (rs1800497) polymorphism. Whereas in one cluster only men with the A1− variant of the DRD2 Taq1A (rs1800497) polymorphism were present, another cluster included only women with mostly the A1− variant of DRD2 Taq1A (rs1800497) polymorphism. The third cluster mainly comprises the A1+ variant of male patients. Furthermore, standardized medication response of the three identified clusters significantly differed, i.e. men without DRD2 Taq1A (rs1800497) risk alleles (A1− variants) have a higher standardized medication response in comparison to the two other patient groups (p < 0.0001; cf. Fig. 1b). Additionally, symptom onset side significantly differed between subgroups. A summary of the cluster characteristics is presented in Table 2.

**Table 2:** Significant cluster characteristics.

	Cluster 1	Cluster 2	Cluster 3
Gender	Women only	Men only	Mostly men
Symptom onset left	Equal	Less left	More left
Disease progression	Low	Fast	Low
Standardized medication response	Lower	High	Lower
DRD2 Taq1A (rs1800497) polymorphism	Mainly negative	All negative	Mainly positive

Overview of significant cluster characteristics.

### Multiple linear regression model

The consecutive regression analysis revealed an optimal model for cluster 1 [including (i) symptom onset side; (ii) tremor-/akinetic-rigidity score, (iii) DRD2 Taq1A (rs1800497) polymorphism, (iv) standardized medication response, (v) interaction of disease progression OFF and standardized medication response] to explain adjusted levodopa equivalent dosage (aLED) with a significant regression equation (F (5,20) = 11.843, p < 0.0001) and an adjusted R^2^ = 68%. Regression analysis of cluster 2 yielded in an optimal model [including (i) disease progression OFF, (ii) standardized medication response, (iii) interaction between disease progression OFF and standardized medication response] to predict aLED (F (3,30) = 3.854, p = 0.0019) with an adjusted R^2^ of 21%. For cluster 3 no significant regression model of aLED could be determined. However, the model explaining the highest amount of variation is based on a single variable (standardized medication response) with adjusted R^2^ = 8.4% (F (1,29) = 3.763, p = 0.062).

## Discussion

We demonstrate the feasibility of a clustering approach resulting in three distinct iPD patient subgroups. Extending earlier studies, describing motor and non-motor characteristics in their clustering approaches, we also considered parameters for (1) gender and (2) DRD2 Taq1A (rs1800497) polymorphism. The results of our analyses suggest, that these additional features might also have an impact on the development of Parkinson’s disease affecting the treatment response to dopamine or dopamine equivalents.

An extensive body of literature exists on the association of gender and iPD (for review see Picillo et al.^23^). A clear trend of a 1.5 times higher incidence of iPD in men is well described^14^; here the neuroprotective influence of estrogen, the genetically determined structural and functional cerebral differences as well as environmental factors are stated as putative mechanisms. Since two out of three clusters consisted only of men or women, gender seems to be a highly predictive factor in iPD and should be generally considered in the analysis of parkinsonian data.

As recently shown in Pelzer et al.^24^, DRD2 Taq1A (rs1800497) polymorphism seems to have a neuroprotective influence on neurons in the nigro-striatal and satellite systems for patients with Parkinson’s disease. Besides, the current study emphasizes an impact of DRD2 Taq1A (rs1800497) polymorphism also on the treatment response to dopaminergic drugs: (1) We were able to predict the daily levodopa intake of the individual patients in cluster 1 with very high significance. (2) Our findings, especially in cluster 2, agree with previous studies^25^, describing a rapid-disease progression in patients with right-dominant symptom onset compared to those with left-sided symptom onset. All patients within this group had the A1− variant of the DRD2 Taq1A (rs1800497) polymorphism. (3) Although we could not identify any significant model predicting the daily levodopa dosage for patients within cluster 3, the characteristic of this cluster is of notable interest. All patients included in this group are carriers of the A1+ variant in DRD2 Taq1A (rs1800497). Current understanding from murine models report an association between expression of ANKK1, where the DRD2 Taq1A (rs1800497) is associated with, and the dopaminergic system functioning in the brain^26^. For instance, while an increase in ANKK1 mRNA levels in the striatum was found after D1R-like stimulation (with SKF38393), the inverse reaction was observed after D2 receptor stimulation (with 7-OH-DPAT)^27^. Hence, differences in the reaction on receptor levels are influenced by the diversity of gene polymorphisms in a complex manner. A recent paper investigating DRD2 Taq1A (rs1800497) polymorphism reported an influence of rare gene variants on the reaction to dopaminergic drugs^28^. Accordingly, differences in the reaction to dopaminergic drugs might be explained by the diversity in the DRD2 Taq1A (rs1800497) polymorphism and the expression in the brain itself. Next to direct alterations on receptor levels, gene polymorphisms are an influencing factor on the brain structure. An interplay between basal ganglia connectivity and D2 receptor availability affects the clinical presentation and medication response of parkinsonian patients^24^. A further sub-differentiation of DRD2 Taq1A (rs1800497) polymorphism (including the anatomical distribution, the percentage of polymorphism in the individual and the sequence variation) might be necessary to better understand the different reactions on different dopaminergic drugs (e.g. dopamine agonists or levodopa).

An altered anatomical background in basal ganglia loops for right- or left-affected parkinsonian patients has already been confirmed in a recent support vector machine analysis^29^. In this study right-dominant expression of iPD has been connected to faster disease progression^30^, indicating a different degeneration pattern with altered neuronal firing rates in oscillatory networks. However, the role of the DRD2 Taq1A (rs1800497) polymorphism in these different clinical situations is still unclear. Other studies investigating the association of iPD with the DRD2 Taq1A (rs1800497) genotype published contradictory findings. Whereas one study could show an increased risk of motor fluctuations^31^, another study could not state any influence of DRD2 Taq1A (rs1800497) polymorphism on dyskinesia^32^. An effect of DRD2 Taq1A (rs1800497) polymorphism on structural brain connectivity in patients with the wild type (A1− variant) compared to the risk type (A1+ variant) has been shown in Pelzer et al.^24^. This correlation of structural changes in brain connectivity due to DRD2 Taq1A (rs1800497) polymorphism can further be linked to differences in the treatment response of individual genotypes. Hence, it might explain differences in the development of side effects like motor fluctuations in Parkinson’s disease. Herewith these results indicate the relevance of the DRD2 Taq1A (rs1800497) polymorphism for investigating dopaminergic side effects in future studies.

In contrast to previous studies identifying subtypes of Parkinson’s disease^8,12^, we ignored non-motor scales and only focused on the implementation of parameters usually assessed in clinical routine (like age, gender, symptom onset side, motor scales, levodopa dosage per day). Beside the described encouraging results some limitations of the study have to be mentioned. One important point is the missing replication cohort in order to externally validate the study. Although the number of 91 patients seems high, the heterogeneity of the symptoms of the parkinsonian patients and the number of variables weakens the statistics and explanatory power. Hence, larger samples with a higher number of patients should be included in future analyses.

Besides increasing the sample size, the clustering process ultimately benefits from s sets of parameters, suited to build computational phenotypes. Features from these parameters may be derived from neuroimaging (e.g. anatomical and diffusion magnetic resonance imaging) as well as clinical tests on non-motor symptoms; also neuropathological findings or gene polymorphisms might enhance predictability about the treatment response in Parkinson’s disease. In conclusion, we consider these results as a major step to further establish predictive clinical models of iPD. The integration of more basic variables and the combination with computational algorithms could provide clinicians with reliable information to develop more individually adapted therapies to relief parkinsonian symptoms more efficiently, while reducing potential side effects. In future approaches ‘big data’ acquisitions suited for phenotyping “the parkinsonian patient” are, however, indispensable. Until then, reliably predicting the individual course of the disease in such a heterogeneous disease pattern remains preliminary. With this study, we focus attention on considering also some more basic mechanistic features influencing disease progression and therapeutic response.

## Supplementary Information

Supplementary Information.
