# Supplementary material for: Clustering of Parkinson subtypes reveals strong influence of DRD2 polymorphism and gender
Source: Sci Rep. 2022 Apr 11;12:6038. doi: 10.1038/s41598-022-09657-0 (PMC9001640; doi:10.1038/s41598-022-09657-0)
Supplement: Supplementary file 1 — Supplementary Information. [file 41598_2022_9657_MOESM1_ESM.docx]

**SUPPLEMENTARY INFORMATION**

**Patients and methods**

**Patients.** We included 118 idiopathic Parkinson’s disease (iPD) patients, who fulfilled the diagnosis criteria according to the “Guidelines of the German society of neurology” (https://www.dgn.org).

To give written consent, only patients fluent in German were included in the study. Further, these patients had to be able to tolerate a dopaminergic withdrawal up to 72-hours.

Exclusion criteria were severe psychiatric and internal diseases, severe structural brain changes (such as ischemic or hemorrhagic infarctions, brain tumors, epilepsy, atypical or secondary Parkinson’s syndrome), other basal ganglia diseases (e.g. dystonia, essential tremor) or deep brain stimulation.

Next to the recruitment in the outpatient clinic of the University Hospital Cologne, patients were acquired from the wards of the neurological department and the Cologne Private Practice Network, a cooperation of the University Hospital and ambulant working neurologists. Hereby a representative cohort of German iPD patients had been obtained.

Gender, age at conduction of study, age at disease onset, disease duration, side of symptom onset and adjusted levodopa equivalent dosage (aLED) were recorded. aLED was calculated following the standard conventions^1^. Disease onset was defined as the date of diagnosis by a neurologist, as documented in the medical report. Considering the complete cohort of 118 patients, 71% were male and 49% had the first Parkinsonian symptoms on the left body side. Their mean age at the time of the study begin was 64 years with an average age of symptom onset of 58 years (cf. Supplementary Table S1 & S2). Due to varying disease durations, we decided to exclude patients suffering less than three years to account for fine-tuning of medication doses. Consequently, study sample size has been reduced to 91 patients with mean disease duration of 7.4 (+/- 4.35) years. Further details can be found in Supplementary Table S1 and S2.

**Clinical Assessment.** The motor part of UPDRS (part III, items 18 – 31) was assessed and videotaped in both medication ‘OFF’ and ‘ON’ state^2^. Although exemplary inter-rater reliability was high, we chose to conduct video rating by a single neurologist (S.S.) to standardize UPDRS-ratings. To reduce prolonged medication effects, dopamine agonist medication was paused 72h before the OFF state measurement for all patients; levodopa and all other parkinsonian medication was stopped 12 hours before. The ‘ON’ state was defined as the patient’s motor condition 60 – 120 minutes after the application of 200/50mg fast acting levodopa/carbidopa (Madopar LT®).

Based on the UPDRS III measurements, additional study parameters expressing disease progression and medication response were derived. In order to calculate disease progression, we adjusted the UPDRS III ‘OFF’ score by the patient’s disease duration. This should best reflect the current clinical status of the patient. Medication ‘ON’ states might be influenced by experience with the medication, individual medication resorption, interactions or possible side effects. Medication response is defined as $1-\frac{\text{UPDRS III ON}}{\text{UPDRS III OFF}}$. Further details can be found in Supplementary Table S3. All classifications of motor subscores and quotients were carried out according to Spiegel et al. ^3^.

**Genotype polymorphism analysis.** The DRD2 Taq1A (rs1800497) polymorphism is located in the ankyrin repeat and protein kinase domain-containing protein 1 (ANKK1) gene, downstream from the dopamine receptor D2 (DRD2) gene and therefore commonly used for D2 genotyping^4^. Genotypes A/A and A/G account as risk type carrying the A1 risk allele, and thereinafter referred to as A1+ carriers. Those not carrying this allele (G/G; wild type) are denoted as A1-. Individuals carrying the minor alleles show diminished striatal D2 density^5^ in comparison to those who do not. For DRD3 Ser9Gly (rs6208) receptor polymorphism, the C minor allele was analyzed, in which the amino acid serine was substituted by glycine. These minor-allele carriers with C/C and C/T genotype are referred as the risk type (DRD3+) and T/T genotype are named as the wild type (DRD3-).

The DNA was isolated from peripheral blood samples using QIAamp DNA Blood Mini Kit (#51106, QIAGEN) according to the manufacturer's instructions. An ND-1000 UV/Vis-Spectrophotometer (Peqlab) assessed concentration and quality of the DNA. SNP genotyping for DRD2 Taq1A (rs1800497) and DRD3 Ser9Gly (rs6208) receptor polymorphism was performed with 20ng of DNA in triplicates using allelic discrimination assays (TaqMan SNP Genotyping Assays, Applied Biosystems by Invitrogen). A 7900HT Fast Real-Time PCR System (Applied Biosystems) was used for genotyping PCR. The obtained fluorescence data was computed with Sequence Detection Software version 2.3 (Applied Biosystems).

**Clustering method.** To consider core parameters of iPD motor symptoms and account for the impact of gender as well as dopamine receptor polymorphisms at the same time, we included the following variables: gender, age at onset, symptom onset side, disease progression OFF, standardized medication response, DRD2 Taq1A (rs1800497) and DRD3 Ser9Gly (rs6208) polymorphism as well as tremor-/akinetic-rigid score. To facilitate the usage of continuous and dichotomous variables in one clustering model, all parameters were standardized by computing z-scores and further transformed into a common variable space using constant-shift-embedding^6^. Similar to singular value decomposition this approach allows estimating the amount of variance explained by each component and can thus be applied for dimension reduction. In this implementation we only included components explaining 99.9% of the variance in the data, resulting in 8 features. Based on these transformed parameters kmeans-clustering^7^ was utilized to identify subclasses within our patient cohort. The optimal cluster number was then assessed by validating all clusterings using the Calinski-Harabasz criterion^8^ (see Supplementary Figure S2). An illustration of our results can be found in Figure 1A in the main article.

After conducting the cluster analysis, we performed post-hoc analysis for each variable. Depending on the variable characteristic (dichotomous or continuous) as well as their distribution (parametric or non-parametric), we used Chi-squared test, ordinary one-way ANOVA or Kruskal-Wallis tests, respectively. Subsequent False Discovery Rate (FDR; q=5%) was applied to adjust p-values for multiple comparisons. Generally, we considered p-values below 0.05 as significant. The variables gender, symptom onset, disease progression OFF, standardized medication response as well as DRD2 Taq1A (rs1800497) polymorphism showed significant differences among the identified subclasses.

**Regression method.** In a next step, multiple linear regression was employed to model the adjusted LED (i.e., the LED normalized by individual disease duration of each patient) and allow for characterizing the identified clusters. Therefore, the most predicting regression models were identified based on their second-order Akaike information criterion^9^ using the R-package MuMIn (<https://CRAN.R-project.org/package=MuMIn>^10^)

For cluster 1 we found the best model fit for explaining the adjusted levodopa equivalent dosage (aLED) with the following predictors: (i) symptom onset; (ii) tremor-/akinetic-rigidity score, (iii) DRD2 Taq1A (rs1800497) polymorphism, (iv) standardized medication response (v) interaction of DRD2 Taq1A (rs1800497) polymorphism and standardized medication. The identified regression model was highly significant with (F (5,20) = 11.843, p < 0.0001) and an adjusted R^2^=68%. For cluster 2 we identified the most predicting model including (i) disease progression OFF, (ii) standardized medication response, (iii) the interaction between disease progression OFF and standardized medication response (F (3,30) = 3.854, p = 0.019) yielding an adjusted R^2^ of 21%. For cluster 3 the most predicting, although non significant, model is based on (i) standardized medication response only (adjusted R^2^ of 8,4% F (1,29) = 3.763, p=0.062).

**Supplementary Tables**

|  | full cohort | study cohort |
| --- | --- | --- |
| Number of patients | 118 | 91 |
| Number of men | 84 (71%) | 62 (68%) |
| Age at onset | 58 (±10) | 58 (±10) |
| Symptom onset side left | 58 (49%) | 44 (48%) |
| Disease progression OFF | 7 (±5) | 5 (±2) |
| Medication response | 0.42 (±0.20) | 0.41 (± 0.20) |
| Standardized medication response | 0.12 (±0.14) | 0.07 (±0.05) |
| DRD2 Taq1A (rs1800497)  Risk type (A/A or A/G) | 40 (34%) | 27 (30 %) |
| DRD3 Ser9Gly (rs6208)  Risk type (C/C or C/T) | 48 (41%) | 36 (40%) |
| Tremor-/akinetic-rigid score | 0.3 (± 0.6) | 0.3 (± 0.5) |

Table S1: **Variables included in the clustering approach.** Age at onset is given in years and disease progression is the UPDRS III OFF score adjusted by the patient’s disease duration. Medication response is given in percentage terms. Continuous variables are displayed as mean (± standard deviation) and dichotomous variables are given as absolute numbers (and its percentage).

|  | full cohort | study cohort |
| --- | --- | --- |
| age | 64 (± 9) | 65 (± 9) |
| disease duration (in years) | 6 (± 5) | 7 (± 4) |
| Levodopa equivalent daily dose (in mg) | 577 (± 383) | 668 (± 383) |
| UPDRS III OFF medication | 29 (± 12) | 31 (± 11) |
| UPDRS III ON medication | 17 (± 9) | 19 (± 9) |

Table S2: **Variables not included in the clustering approach.** Age and disease duration are given in years. The amount of levodopa equivalent daily dose is indicated in milligram. Continuous variables are displayed as mean (± standard deviation).

|  | Formula |
| --- | --- |
| UPDRS III OFF medication | $\sum UPDRS III items 18 - 31 after 12h/72h medication pause$ |
| UPDRS III ON medication | $\sum UPDRS III items 18 - 31 after 200/50mg levodopa/carbidopa$ |
| disease progression OFF | $\frac{UPDRS III OFF}{disease duration}$ |
| medication response | $\left( 1-\frac{UPDRS III ON}{UPDRS III OFF} \right)$ |
| Standardized medication response | $\frac{medication response}{disease duration}$ |
| Tremor-score OFF | μ(UPDRS III OFF items 20 & 21) |
| Akinetic-rigid score OFF | μ(UPDRS III OFF items 18, 19, 22, 27 – 31) |
| Tremor-/Akinetic-rigid score | $\frac{Tremor-score OFF}{Akinetic-rigid score OFF}$ |

Table S3: **Description of variables included in the clustering approach.** UPDRS is the abbreviation for Unified Parkinson’s Disease Rating Scale. A tremor-/akinetic-rigid score below 0.5 is considered akinetic-rigid type, in the range 0.5 – 2 is considered mixed type and above 2 is a tremor-dominant type.

| Variables | Cluster 1 | Cluster 2 | Cluster 3 | Test statistics | p-value |
| --- | --- | --- | --- | --- | --- |
| number of patients | 26 | 34 | 31 |  |  |
| number of men | 0 (0%) | 34 (100%) | 28 (90%) | $\chi^{2}$ (2)=78.52 | **< 0.0001** |
| age at onset | 60 (± 9) | 57 (± 9) | 56 (± 11) | $F\left( 2, 88 \right)= 1.253$ | 0.29 |
| Symptom onset side left | 12 (46%) | 11 (32%) | 21 (68%) | $\chi^{2}(2)=8.202$ | **0.017** |
| disease progression OFF | 4 (± 2) | 5 (± 3) | 4 (± 2) | $F\left( 2, 88 \right)= 11.23$ | **<0.0001** |
| Standardized  medication response | 0.066  (± 0.04) | 0.097  (± 0.04) | 0.047  (± 0.03) | H(2)=23.08 | **< 0.0001** |
| DRD2 Taq1A (rs1800497)  Risk type  (A/A or A/G) | 4 (15 %) | 0 (0%) | 23 (74%) | $\chi^{2}$ (2)=46.34 | **< 0.0001** |
| DRD3 Ser9Gly (rs6208)  Risk type  (C/C or C/T) | 9 (35%) | 13 (38%) | 14 (45%) | $\chi^{2}$ (2)=0.70 | 0.71 |
| T-/AR-score | 0.0 (± 0.4) | 0.0 (± 0.1) | 0.15 (± 0.1) | $H\left( 2 \right)=4.606$ | 0.1 |

Table S4: **Statistical cluster comparison.** Dichotomous variables, i.e., gender, symptom side of onset, DRD2 Taq1A (rs1800497) and DRD3 Ser9Gly (rs6208) are stated as absolute numbers (sample percentage) and were compared using a Pearson’s Chi-Squared Test. Ages at onset and medication response are stated as mean (± standard deviation) and were compared using an ordinary one-way ANOVA due to parametric distribution. Standardized medication response and T-/AR-score are stated as median (± standard error of the mean) and were compared by Kruskal-Wallis test due to nonparametric distribution. All p-values below 0.05 are considered to be significant and are printed in bold.

**Supplementary Figure**

**
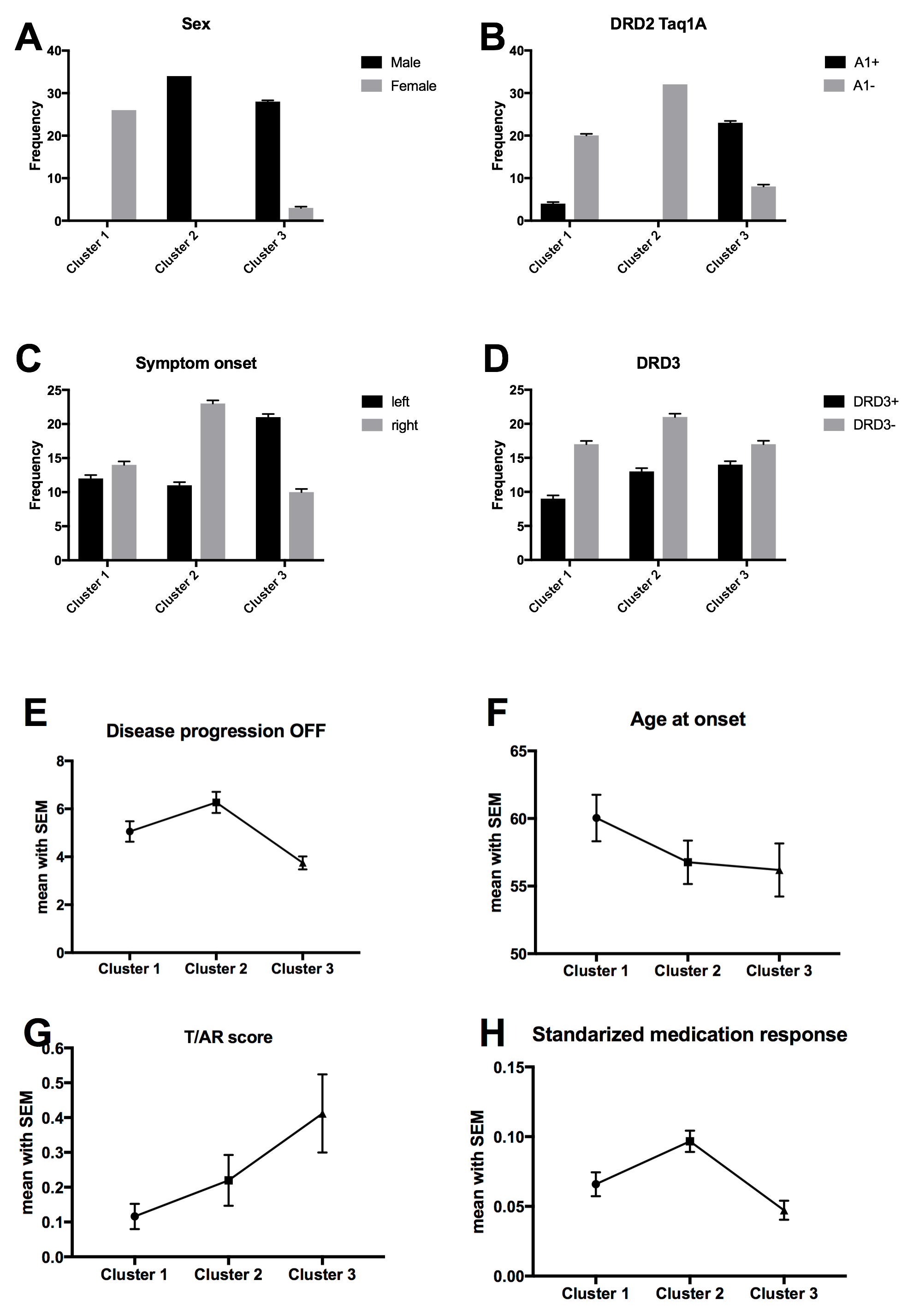
**

Figure S1: **Cluster comparison.** Overview of all cluster characteristics. (A) Bar plots of men and women within each cluster, respectively. (B) Bar plots of DRD2 Taq1A (rs1800497) polymorphism within each cluster. (C-D) Bar plots of left and right symptom onset side as well as DRD3 Ser9Gly (rs6208) polymorphism within each cluster, respectively. (E) Disease progression OFF displayed as connected mean with standard error of means within each cluster. (F-H) Age at onset and T-/AR-score and standardized medication response are displayed as connected mean with standard error of means within each cluster, respectively.


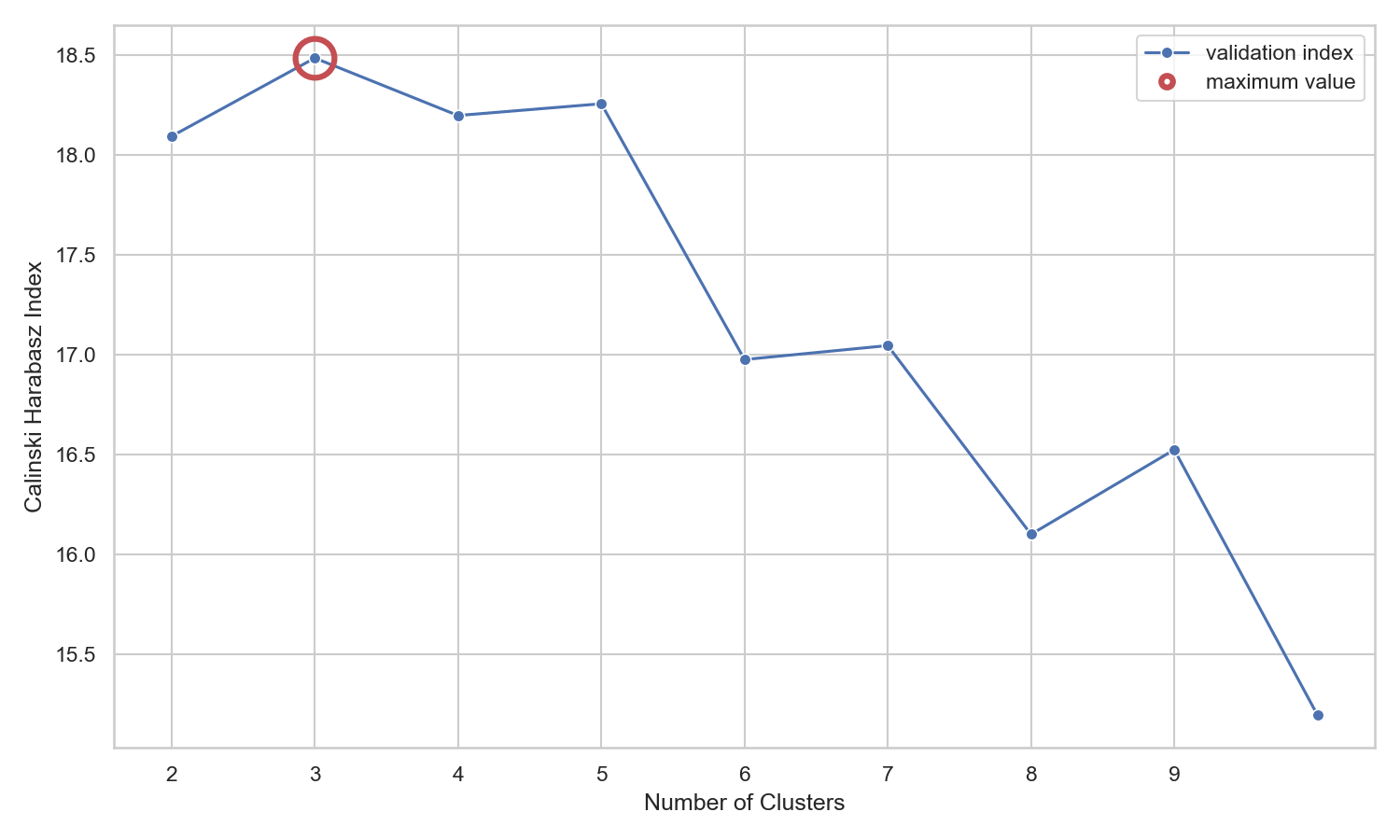


Figure S2: **Calinski-Harabasz validation.** Calinski-Harabasz validation index for cluster numbers 2 to 10 (red circle represents optimal clustering solution).

**References:**

1. Tomlinson, C.L., et al. Systematic review of levodopa dose equivalency reporting in Parkinson's disease. Movement Disorders **25**, 2649-2653 (2010).

2. Fahn S, E.R., UPDRS program members. Unified Parkinsons Disease Rating Scale. Floram Park, NJ: Maximilian Healthcare Information **2**, p. 153-163 (1987).

3. Spiegel, J., et al. Striatal FP-CIT uptake differs in the subtypes of early Parkinson's disease. Journal of Neural Transmission/General Section JNT **114**, 331-335 (2007).

4. Neville, M.J., Johnstone, E.C. & Walton, R.T. Identification and characterization of ANKK1: a novel kinase gene closely linked to DRD2 on chromosome band 11q23.1. Human mutation **23**, 540-545 (2004).

5. Jönsson, E.G., et al. Polymorphisms in the dopamine D2 receptor gene and their relationships to striatal dopamine receptor density of healthy volunteers. Molecular psychiatry **4**, 290-296 (1999).

6. Roth, V., Laub, J., Kawanabe, M. & Buhmann, J.M. Optimal cluster preserving embedding of nonmetric proximity data. IEEE Transactions on Pattern Analysis and Machine Intelligence **25**, 1540-1551 (2003).

7. Bishop, C.M. Pattern Recognition and Machine Learning, (Springer, 2016).

8. Caliński, T. & Harabasz, J. A dendrite method for cluster analysis. Communications in Statistics - Theory and Methods **3**, 1-27 (1974).

9. Hurvich, C.M.a.T., C.-L. Regression and time series model selection in small samples. Biometrika **76**, 297–307 (1989).

10. Burnham, K.P.a.A., D. R. Model selection and multimodel inference: a practical information-theoretic approach. Springer-Verlag (2002).
